# Focus on Biochemical and Clinical Predictors of Response to Immune Checkpoint Inhibitors in Metastatic Urothelial Carcinoma: Where Do We Stand?

**DOI:** 10.3390/ijms21217935

**Published:** 2020-10-26

**Authors:** Giandomenico Roviello, Martina Catalano, Stefania Nobili, Raffaella Santi, Enrico Mini, Gabriella Nesi

**Affiliations:** 1 Department of Health Sciences, Section of Clinical Pharmacology and Oncology, University of Florence, Viale Pieraccini, 6, 50139 Florence, Italy; stefania.nobili@unifi.it (S.N.); enrico.mini@unifi.it (E.M.); 2 School of Human Health Sciences, University of Florence, Largo Brambilla 3, 50134 Florence, Italy; marti_cat@yahoo.it; 3 Department of Pathology, Careggi University Hospital, University of Firenze, 50139 Firenze, Italy; raffaella.santi@unifi.it; 4 Department of Health Sciences, University of Florence, Section of Pathological Anatomy, University Hospital of Florence, 50139 Florence, Italy; gabriella.nesi@unifi.it

**Keywords:** PD-1, PD-L1, bladder cancer, biomarkers

## Abstract

Urothelial bladder cancer is one of the most lethal cancers worldwide with barely 5% five-year survival in patients with metastatic disease. Intravesical immunotherapy with *Bacillus Calmette-Guérin* and platinum-based chemotherapy are currently the standard of care for non-muscle invasive and advanced or metastatic urothelial cancer (mUC), respectively. Recently, a subset of patients with locally advanced or mUC has shown to be responsive to immune checkpoint inhibitors (ICIs), e.g., the anti-cytotoxic T-lymphocyte-associated protein 4 and programmed cell death -1/programmed death-ligand1 (PD-1/PD-L1) antibodies. Due to the relevant clinical benefit of immunotherapy for mUC, in 2016, the United States Food and Drug Administration (FDA) approved five immunotherapeutic agents as second-line or first-line treatments for patients with advanced bladder cancer who did not profit from or were ineligible for standard therapy. In this review, we discuss the role of immunotherapy in bladder cancer and recent clinical applications of PD-1/PD-L1 blockade in mUC. Furthermore, we evaluate a variable response rate to ICIs treatment and outline potential biomarkers predictive of immunotherapy response.

## 1. Introduction

Bladder cancer is the tenth most commonly diagnosed tumor worldwide with high morbidity and mortality of up to 165,000 deaths per year [1]. Several risk factors for bladder cancer have been extensively investigated with tobacco smoke and occupational exposure to carcinogens being associated with a four-fold increased risk [2,3,4]. Bladder cancer is a heterogeneous group of tumors of which urothelial carcinoma is the most common histological subgroup.

Approximately 70% of bladder carcinomas manifest as non-muscle invasive disease requiring only local treatment. However, on account of their high recurrence rate, it may be necessary to consider systemic therapy. Contrariwise, muscle-invasive and metastatic bladder carcinomas entail multimodal treatments including surgery and chemotherapy [5]. The treatment algorithm for advanced bladder cancer is rapidly evolving and novel effective therapies are replacing standard chemotherapy. In recent years, immunotherapy has taken on a prominent role in oncology as a first-line monotherapy in numerous tumor types, including metastatic urothelial cancer (mUC), or, more frequently, in addition to conventional cancer treatments (i.e., surgery, chemotherapy, and radiotherapy) [6]. Cancer immunotherapy is an artificial stimulation of the immune system, boosting effector mechanisms while suppressing inhibitory mechanisms in order to counteract tumor development [6,7]. Since 1980, several types of immunotherapies have been developed, such as immune check point inhibitors (ICIs), T-cell transfer therapy, monoclonal antibodies, vaccines, and immune system modulators [7,8].

To date, intravesical therapy with *Bacillus Calmette-Guérin* (BCG) or chemotherapy are the mainstay of treatment following transurethral bladder tumor resection (TURBT) in non-muscle invasive carcinomas [9,10,11]. BCG causes a strong innate immune response that leads to long-term adaptive immunotherapy [12,13]. The BCG vaccine has been used to treat bladder cancer for decades but its mechanism of action is not yet fully understood [9,12].

ICIs have revolutionized the treatment of mUCs, despite merely 20–30% of patients showing clinical response, and an even smaller number achieving a durable response of more than two years [14].

The mechanism behind the dissimilar reactions to ICIs among mUC patients has not been thoroughly elucidated, and variation in the response rate to these drugs for different tumor types have encouraged the search for biomarkers. In this review, we focus on the current status of immunotherapy in bladder cancer, highlighting advances in the identification of genomic and clinical biomarkers for predicting an ICI response.

## 2. Immune Checkpoints

Immune checkpoints are regulators of the immune system. These pathways are crucial for self-tolerance, which prevents the immune system from attacking cells indiscriminately. Although, at least in the initial phases of carcinogenesis, tumor cells should be detectable by the immune system and, thus, targetable and destroyed. They can escape immune recognition and subsequent destruction through various resistance mechanisms (e.g., local immune evasion, induction of tolerance, and systemic disruption of T cell signaling). In addition, during the immune editing process, the recognition of malignant cells exerts a selective pressure on developing tumors and leads to the growth of less immunogenic and more apoptosis-resistant tumor cells [15].

**Figure 1:**
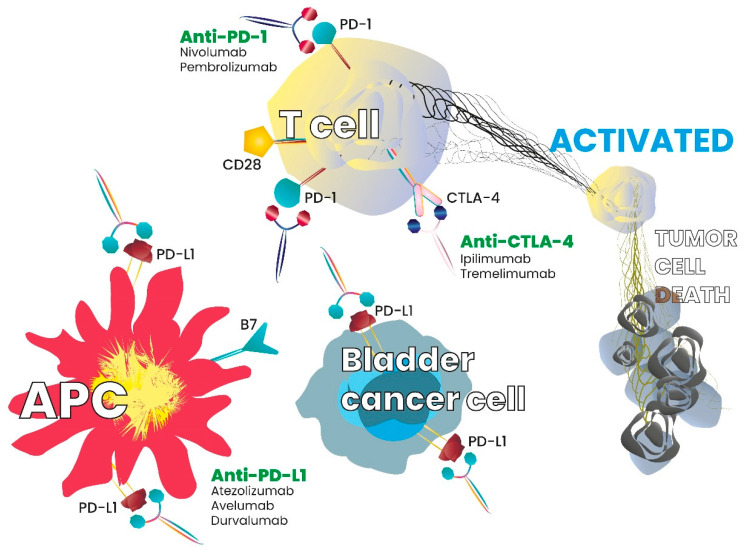
Checkpoint inhibitors and interaction with the immune system or tumor cells.

Some cancers are known to overcome immune system attacks by stimulating immune checkpoint targets [16]. Inhibitory checkpoint molecules, such as cytotoxic T-lymphocyte-associated-4 (CTLA-4) and programmed cell death-1 (PD-1), are receptors expressed on the surface of cytotoxic T cells that, respectively, interact with their ligands CD80/CD86 and the programmed death ligand-1 (PD-L1) [17,18,19]. Inhibitory checkpoint molecules are, therefore, targets for cancer immunotherapy, as antibodies directed against them block the downregulation signal and increase the T cell response. Checkpoint inhibitors and interaction with the immune system or tumour cells is summarized in Figure 1.

### 2.1. PD-1/PDL-1 Pathway in Normal Cells

PD-1 (CD279) is a 288-amino acid type I transmembrane protein receptor, predominantly expressed on antigen-experienced memory T cells in peripheral tissues, and, to a lesser extent, on B cells and macrophages [20]. Encoded by the *PDCD1* gene, it is homologous to the CD28 family of protein receptors. Structurally, PD-1 consists of an immunoglobulin V (IgV)-like extracellular domain, a trans-membrane domain, and a cytoplasmic tyrosine kinase domain that, upon phosphorylation, negatively regulates T cell receptor (TCR) signals by phosphorylating Src homology phosphatase-1 (SHP-1) and SHP-22 [20].

PD-L1 (B7-H1 or CD274) is one of the two ligands of PD-1 and a member of the B7 family of type I transmembrane protein receptors [21]. It is a 290-amino acid protein receptor encoded by the *CD274* gene and composed of two extracellular domains, IgV and IgC, a transmembrane domain, and a cytoplasmic domain.

PD-1 expression is induced upon TCR/antigen-loaded major histocompatibility (MHC) and CD28/B7 interactions [21]. When PD-L1 binds to PD-1, the PD-1 axis decreases the T cell response in several ways primarily by cytokine production [21,22]. PD-1 is also highly expressed on regulatory T cells that, following activation of PD-1/PD-L1 signaling, can proliferate and inhibit the immune response by expressing the transcription factor FOXP3, a lack of effector cytokine expression (e.g., IFNγ), and production of inhibitory cytokines (e.g., TGFβ, IL-10, and IL-35) [23,24].

### 2.2. PD-1/PDL-1 Pathway in Tumor Cells

Since the PD-1 signal can be exploited by tumor cells to evade an anti-cancer immune response, the PD-L1/PDL-1 pathway is the most broadly studied immune checkpoint in cancer, including urothelial cancer [14].

PD-1 is highly expressed in tumor-infiltrating lymphocytes (TILs) in solid tumors, while PD-L1 is constitutively expressed and/or upregulated in many types of cancer cells [25,26]. PD-L1 upregulation is primarily due to innate and adaptive immune resistance. The interaction of PD-1/PD-L1 in the tumor microenvironment can induce resistance to cytotoxic T cell (CD8+)-mediated elimination of tumor cells by promoting cancer development and progression [26,27].

ICIs prevent the receptor and the ligand from binding to each other, thereby destroying checkpoint signaling [17,28]. PD-1/PD-L1 inhibitors are antibodies that block either of these two molecules, resulting in T cell activation [17]. As far as mUC is concerned, ICIs were initially employed as second-line therapy, but, over the last few years, they have also acquired an increasingly important role as first-line therapy.

### 2.3. Anti PD-1 Antibodies

Pembrolizumab and nivolumab were the first two anti PD-1 monoclonal antibodies to receive the United States Food and Drug Administration (FDA) approval.

Pembrolizumab is a humanized IgG4/Kappa monoclonal antibody approved in May 2017 as second-line treatment for bladder cancer, based on results from phase III trials [29,30,31]. According to the European Medicines Agency (EMA), pembrolizumab may also be used as first-line therapy in UC [32]. In the phase III KEYNOTE-045 trial, pembrolizumab was compared with conventional chemotherapy in 542 patients affected by mUC, previously treated with platinum-based chemotherapy. Patients were randomized to receive either pembrolizumab or the physician’s choice among docetaxel, paclitaxel, and vinflunine. Overall survival (OS) and progression-free survival (PFS) were co-primary endpoints. Pembrolizumab was associated with significantly longer OS (by 2.9 months) and a better safety profile than the other drugs [33]. In particular, the median OS in the total population was 10.3 (8.0 to 11.8) months in the pembrolizumab group, as compared with 7.4 months (95% CI, (6.1% to 8.3 95% confidence interval)) in the chemotherapy group (hazard ratio (HR) for death, 0.73). Pembrolizumab was also tested in the phase II KEYNOTE-052 trial on bladder cancer patients who were ineligible for cisplatin-based chemotherapy. PD-L1 expression levels in tumor biopsies were one of the inclusion criteria and the primary endpoint was the objective response rate (ORR). The ORR was 24% at data cutoff and an excellent durable response rate was recorded in 89% of responders [34]. In addition, median PFS was 2 months (95% CI 2–3) with a 6-month overall survival in 67% of all patients.

Nivolumab is a human IgG4 monoclonal antibody, approved by the FDA in February 2017 for advanced bladder cancer [35]. In a phase I/II trial, nivolumab was tested on patients with locally advanced or metastatic platinum-pretreated UC [36]. The ORR (primary endpoint) reached 24.4%. A median OS of 9.7 months was recorded and 21.8% of patients experienced grade 3–4 toxicity. No significant correlation between OS and PD-L1 overexpression was observed.

In the CHECKMATE275 phase II study, performed on a larger sample of patients with advanced UC, the ORR (primary endpoint) was 19.6% and median survival was 8.47 months. Patients with PD-L1 over-expression responded differently to treatment from patients with low PD-L1 expression. The ORR was 28.4%, 23.8%, and 16.1% in patients with PD-L1 ≥ 5%, PD-L1 ≥ 1%, and PD-L1 < 1%, respectively. Grade 3–4 toxicity occurred in 18% of patients [37].

### 2.4. Anti PD-1 Antibodies Conjugations

More recently, a multi-cohort study (NCT03288545) evaluated the safety/activity of enfortumab vedotin (EV), an antibody-drug conjugate, which delivers the microtubule-disrupting agent, monomethyl auristatin E (MMAE), to cells expressing Nectin-4, plus pembrolizumab in first-line cisplatin ineligible patients [38]. EV is highly expressed in UC and has shown activity in previously treated mUC [39]. The primary endpoint was safety and tolerability, while secondary endpoints included dose limiting toxicities, ORR, diagnostic odds ratio (DOR), PFS, and OS. The most common treatment-emergent adverse events (TEAEs) were fatigue (58%), alopecia (53%), and peripheral sensory neuropathy (53%). One patient died as a result of multiple organ failure. With a median follow-up of 11.5 months, the ORR was 73.3% in all patients, including 15.6% complete responses (CRs). In particular, the ORR was 78.6% in PD-L1 high (score ≥ 0%) and 63.2% in PD-L1 low (score < 10%). Median PFS was 12.3 months, while median DOR was not attained. In randomized phase III trials, pembrolizumab is the only ICI to show improved OS when compared with chemotherapy [30].

### 2.5. Anti PD-L1 Antibodies

Atezolizumab is a humanized IgG1 monoclonal antibody targeting PD-L1 [30,40,41]. It was approved by the FDA in 2016 as second-line therapy for patients with advanced bladder cancer [29,42] and, in 2017, received accelerated approval as front-line treatment for patients with locally advanced or mUC ineligible for cisplatin chemotherapy [43].

In a phase I study, atezolizumab was tested in patients with mUC previously treated with platinum-based chemotherapy or within one year from neo-adjuvant chemotherapy, an ORR (primary endpoint) of 26.2%, and a good safety profile with grade 3–4 adverse effects in 19% of patients [41]. An appreciable ORR and a durable response to atezolizumab were also recorded in the phase II IMvigor210 trial. In this trial, atezolizumab was used as first-line in cisplatin-ineligible patients and as second-line after failure of standard chemotherapy. The ORRs were 23% and 15%, respectively. Notably, the ORR in the second-line setting ranging from 8% to 26%, depending on PD-L1 expression in tumor-infiltrating immune cells [44]. In the IMvigor211 phase III randomized trial, patients received atezoli-zumab or the physician’s choice of chemotherapy in a second-line setting [45]. No significant difference in survival emerged when patients were stratified, according to the degree of PD-L1 expression of tumor-infiltrating immune cells. However, duration of the response was longer in the atezolizumab group than in the chemotherapy group (15.9 months vs. 8.3 months).

Durvalumab is a monoclonal IgGIK antibody approved by the FDA in 2017 for bladder cancer treatment [46]. It was tested in a phase I/II study on mUC patients who were divided into two groups acccording to PD-L1expression levels. PD-L1 was defined as positive if either ≥25% of tumor cells or ≥25% of immune cells expressed PD-L1, whereas PD-L1 was defined as negative if both ≥25% of tumor cells and ≥25% of immune cells expressed PD-L1. The ORR was 31% in the group of patients treated without assessing PD-L1 expression levels, while it reached 46% in the PD-L1 positive group [47]. In another phase II study conducted on patients with advanced UC, either ineligible for or previously treated with platinum-based chemotherapy, the ORR was lower (17.8%). It was 27.6% in PD-L1 high patients, and 5.1% in PD-L1 low or negative patients [48].

Finally, avelumab, another IgG1 antibody targeting PD-L1, was approved by the FDA in 2017 for urothelial bladder cancer [30,49]. In the phase I JAVELIN trial, an ORR of 18.2% at a median follow-up of 11 months was reported in 44 mUC patients, previously treated with platinum-based chemotherapy and unselected for PD-L1 expression. Both ORR and PFS (secondary objectives) were significantly higher in patients with ≥5% PD-L1 positive tumor cells than in patients with <5% positive tumor cells [50]. A similar ORR (16%) was seen in the expansion phase of the JAVELIN study with a minor difference between PD-L1 positive and PD-L1 negative subgroups [51]. Recently, avelumab has also shown efficacy as maintenance therapy on completion of platinum-based chemotherapy [52].

### 2.6. CTLA-4 Pathway

Cytotoxic T lymphocyte antigen-4 (CTLA-4) is a surface molecule expressed by active T cells, while its ligands, B7.1 and B7.2, are detected on B lymphocytes’ dendritic cells and macrophages [16]. CTLA-4 is a co-stimulator necessary for activation of T lymphocytes, and transmits inhibitory signals to T cells [53,54,55,56].

Ipilimumab is the main monoclonal anti CTLA-4 antibody, approved for treatment of several cancer types [57]. The safety of this drug in UC patients was investigated by Carthon et al., and a good safety profile emerged, characterized by grade 1–2 adverse events [49]. A phase II study investigated the safety, efficacy, and immunomodulatory effects of gemcitabine and cisplatin plus ipilimumab versus chemotherapy alone in patients with mUC. OS (primary endpoint) did not reach statistical significance. While these results contributed to our understanding of the feasibility of combining chemotherapy with immune checkpoint blockade in mUC [58], studies on the combination of ipilimumab with anti PD-1/PD-L1 blockers are ongoing [59].

## 3. Biochemical and Clinical Predictors for the ICI Response

Treatments based on immune checkpoint inhibition have proved to achieve a durable response in a subset of patients with different types of cancer, including UC. However, most patients administered with these drugs do not show any clinical benefit or experience a long-lasting response. The mechanisms underlying variation in the ICI response among mUC patients are poorly understood. Consequently, it is essential to identify the predictive ICI biomarkers as well as the resistance mechanisms in ICI non-responders. Currently, the only approved predictive biomarkers of ICI response are PD-L1 expression for specific cancers and microsatellite instability-high (MSI-H)/mismatch repair deficiency (dMMR) for tumor agnostic therapy. However, there are several other predictive biomarkers under examination for mUC, including a tumor mutational burden (TMB), gene expression profiles (GEP), TCGA (The Cancer Genome Atlas) profiling, and tumor infiltration lymphocytes (TILs).

### 3.1. PD-L1 Expression

In mUC, PD-L1 expression detected by immunohistochemistry does not reproducibly correlate with the treatment response. Moreover, in studies which correlated PD-L1 expression and response, the negative predictive value of the test used was generally poor and did not allow clinical discrimination between responders and non-responders [34,60,61]. An elevated response rate was observed in high PD-L1 groups treated in second-line with pembrolizumab and durvalumab, or in first-line with avelumab. No relationship between PD-L1 expression levels and OS was observed in patients treated in second-line with nivolumab or pembrolizumab [33,39,47,60]. Therefore, the role of PD-L1 expression as a biomarker for the identification of patients who are likely to benefit from ICI is still controversial. In fact, several issues are arising. First of all, no guideline is available for the evaluation of PD-L1 positivity to select patients for anti-PD-1/PD-L1 treatment. Second, there are different PD-L1 assays for the definition of PD-L1 positivity. Although, all the assays evaluated the membrane expression of PD-L1 by ICH, differences in staining methods or scoring systems, components for the evaluation (tumor cells vs. immune cells) bring discordant results depending on the assay. As a result, it is difficult to compare the effectiveness of ICIs according the PD-L1 positivity across all investigated drugs in the trials. Third, biological challenges such as variation in PD-L1 expression or heterogeneity of PD-L1 expression and technical challenges may influence the PD-L1 expression.

There are several other predictive biomarkers under examination for mUC and mRCC, including tumor mutational burden (TMB), mismatch repair status, gene expression profiles (GEP), TCGA (The Cancer Genome Atlas) profiling, tumor infiltration lymphocytes (TILs), and PD-L2.

### 3.2. TMB and Ne-Oantigens

Single nucleotide variant (SNV) count is defined as the number of exonic non-synonymous mutations per sample, including indels, non-sense mutations, splice site mutations, and non-synonymous mis-sense variants, often referred to as tumor mutational burden (TMB). The mUC is characterized by a high somatic mutation rate pathway, particularly in the Lund genomically unstable (LGU) group and TCGA cluster II group, as shown in a post hoc analysis of the IMvigor210 trial in which the response to atezolizumab was slightly higher in the TCGA cluster II than in other subtypes [43,62,63,64]. Significant associations between the TMB and ICI response have been reported in a variety of tumors, including mUC [43,65]. In mUC, data from IMvigor 210 further suggest that high TMB may not be only prognostic of survival, but potentially predictive of a response [43,44]. Patients in the highest quartile (quartile 4) of TMB had a significantly longer mOS when treated with atezolizumab as compared with those in quartiles 1–3 (*p* = 0.0041) [43]. High TMB patients have also been shown in Checkmate 275 to have a better ORR (31.9% vs. 17.4%, *p* = 0.002) and median PFS (3.02 months vs. 1.87 months) [66]. Recently, high TMB was also found in mUC patients who also harbored ERBB2 (HER2) and ERBB3 (HER3) mutations [67]. The clinical applicability and predictive power of TMB is uncertain [68]. TMB alone does not clearly discriminate all responders from non-responders. In mUC, low TMB does not preclude response nor is a high TMB sufficient to predict a response [69].

Elevated TMB has been correlated with an increased chance of generating immunogenic neo-antigens [70]. True neo-antigen burden, or the number of mutations actually targeted by T cells, may have a stronger relationship with the ICI response than TMB [71,72,73,74,75]. The IMvigor210 trial, through a computational approach combining somatic nucleotide variation data sets [74,76], confirmed the association between high levels of predicted neo-antigens in response to atezolizumab [77], demonstrating this parameter to be a stronger predictor of response than TMB alone.

### 3.3. Copy Number Variant (CNV)

The association between copy number variant (CNV), characterized by homozygous deletions and amplifications (defined as > six copies) [78,79] and ICI response, has recently been analyzed in several cancer types [80]. In mUC, high CNV count was associated with less of a clinical benefit from ICI treatment and poorer PFS, but not with OS. In Nassar et al., homozygous deletion of CDKN2A and CDKN2B was the most common CNV event related to no clinical benefit. CDKN2B was significantly linked to worse PFS and OS, even though correlation with CDKN2B deletions had not been reported in previous studies. Furthermore, analysis of the interaction between SNV and CNV counts and ICI response revealed that patients with high SNV and low CNV benefitted more from ICI therapy than patients with low SNV and high CNV counts.

### 3.4. Microsatellte Instability

Mutation of genes involved in the DNA MMR pathway causes defective DNA damage repair (dMMR), which has been shown to be a predictive biomarker for response to ICIs [81,82]. Microsatellite instability (MSI) in the tumor with dMMR, particularly deleterious mutations, was related to a higher sensitivity to ICI treatment regardless of the tissue of origin [83].

MSI positive cancers are TMB-high tumors in which dMMR generates an elevated load of insertion and deletion mutations [74]. In clinical trials performed in mUC patients treated with nivolumab or atezolizumab, defective DNA damage repair was strongly associated with a response to anti PD-1 and anti PD-L1 agents and survival outcome, proving to be superior to TMB [73]. Conversely, association of DNA MMR with ICI response was lower than with TMB in the analysis of the larger IMvigor210 dataset, which was not restricted to deleterious mutations [68]. However, it is unclear whether these conflicting results may be due to the examination of different mutations in DNA damage repair genes. Finally, a further molecular correlation with ICI response was found in the gene set analysis of patients enrolled in the IMvigor210 trial [68]. Genes related to transforming growth factor-beta (TGF-β) signaling in fibroblasts were found to be enriched in ICI non-responders, and the most common gene expression in the TGF-β pathway, such as *TGF-β1* and *TGF-β2*, were associated with reduced OS [68]. TGF-β is a pleiotropic protein likely to be pro-tumorigenic in advanced cancer due to its role in stromal activation, angiogenesis, and epithelial-mesenchymal transition [84]. In mouse tumor models, co-treatment with monoclonal antibodies against both PD-L1 and TGF-β led to an increase in a CD8+ effector gene expression signature and tumor regression [68].

### 3.5. Gene Expression Profiles (GEP)

Gene expression profiling has demonstrated utility as a predictive biomarker to immunotherapy [85,86,87,88]. The expression of interferon gamma is one of the key biomarkers that has been explored as a predictive biomarker for these agents. The innate and adaptive antitumor response to the abnormal cellular growth begins with the production of interferon gamma by activated T cells, natural killer (NK) cells, and the tumor microenvironment. It can lead to upregulation of both PD-L1 and PD-L2 and also upregulate the expression of immunosuppressive molecules such as IDO1. Thus, interferon gamma is key in protecting the host from developing tumors, but it also facilitates tumor escape mechanisms from the immune system [89]. The gene expression profile (GEP) score was used in the trial that, in the KEYNOTE-052, testing pembrolizumab in first line cisplatin-ineligible advanced urothelial cancer was found to have a significant association between the GEP score and treatment response (*p* < 0.0001) [90]. The GEP score could correctly predict a number of responders greater than PD-L1 CPS cutoff (70 vs. 41 of the 81), but its utility still needs to be validated in large prospective clinical trials.

### 3.6. Tumor Infiltrating Cytotoxic T Lymphocytes (TILs)

ICIs enhance the activity of the adaptive immune system by recruiting CD8 positive cytolytic T cells into the tumor microenvironment [91]. At first in metastatic melanoma, CD8 positive T cell density have been described as a predictive biomarker for response to ICIs [91]. In addition, in mUC, high T cell infiltration and clonality were correlated with the treatment response and increased TILs within the tumor microenvironment, which have been found to correlate with improved disease-free survival and overall survival [91,92]. In a retrospective evaluation of 31 patients with muscle invasive UC, patients with more than 8 CD8 TILs had a longer median survival than patients with less than 8 CD8 cells more than 80 months vs. 13 months, respectively [92]. In IMvigor 210 of atezolizumab in platinum-refractory mUC, the inflammatory CD8 cytotoxic T-cells within the tumor microenvironment was associated with an objective response to atezolizumab (*p* = 0.0265) [86]. These tests, despite the poor handling, could prove to be very useful as biomarkers for our response to ICIs.

### 3.7. Clinical Predictors

The existence of clinical predictors of ICI response was evaluated in mUC patients treated with second-line atezolizumab [93]. A preliminary prognostic model was proposed in IMvigor210 and PCD4989g trials. ECOG PS 1 vs. 0, platelet count, liver metastasis, lactate dehydrogenase levels, anemia, and neutrophil-to-lymphocyte ratio (NLR) were included in the optimal prognostic model for OS. Recently, Nassar et al. examined clinical factors for predicting response to ICI therapy in mUC [71]. Lack of visceral metastasis and NLR < 5 were predictive of clinical benefit. The mechanism behind the association of high NLR and lack of clinical benefit to ICI, previously reported in other studies, is still under debate. Neutrophilia may possibly correlate with neutrophil abundance in tumor tissue, thus contributing to a pro-tumor microenvironment [20]. Furthermore, infiltrating neutrophils are also likely to inhibit T cell activation through PD-L1 expression [94]. These clinical factors, combined with a high single nucleotide variant count, constitute a three-factor model specifically predicting ICI response in mUC, but results should be re-tested in larger and more uniformly treated cohorts.

## 4. Conclusions

Bladder cancer is one of the most common lethal cancers with a high rate of recurrence, often requiring multiple treatments, which lead to the inevitable development of drug resistance. Platinum-based chemotherapy still remains the standard of care for advanced UC. However, immunotherapy seems a promising alternative. In particular, ICIs have gradually emerged as the treatment of choice both in patients who have failed prior platinum-based chemotherapy and, more recently, used as front-line therapy or in combination with platinum-based drugs. However, less than a third of mUC patients respond to ICIs, and an even smaller proportion achieve durable responses. The mechanisms underlying variation in the ICI response among mUC patients are poorly understood, but current knowledge indicates that no single biomarker can identify patients likely to benefit from this immunotherapy. However, ICIs treatments are very expansive and, to date, no data are available in the optimal duration of therapy with ICIs to get the response and, therefore, development of a predictive model that examines the different components affecting tumor-host interactions is mandatory to evaluate the individual contribution of each of these elements for the ICI response in order to avoid excessive expenses. This model will require continuous update and re-evaluation, but could have profound implications in the area of precision immune-oncology, thus maximizing the patient benefit from these therapies.

## Abbreviations

(BCG)	Bacillus Calmette-Guérin
(mUC)	metastatic urothelial cancer
(ICIs)	immune checkpoint inhibitors
(PD-1/PD-L1)	programmed cell death-1/programmed death ligand-1
(FDA)	Food and Drug Administration
(TURBT)	transurethral bladder tumor resection
(CTLA-4)	cytotoxic T-lymphocyte-associated-4
(IgV)	immunoglobulin V
(TCR)	T cell receptor
(TILs)	tumor-infiltrating lymphocytes
(EMA)	European Medicines Agency
(OS)	overall survival
(PFS)	progression-free survival
(ORR)	objective response rate
(MMAE)	monomethyl auristatin E
(EV)	enfortumab vedotin
(DOR)	diagnostic odds ratio
(TEAEs)	treatment-emergent adverse events
(MSI-H)	microsatellite instability-high
(dMMR)	mismatch repair deficiency
